# Aldehyde dehydrogenase 2 alleviates monosodium iodoacetate-induced oxidative stress, inflammation and apoptosis in chondrocytes via inhibiting aquaporin 4 expression

**DOI:** 10.1186/s12938-021-00917-0

**Published:** 2021-08-06

**Authors:** Lingxiao Pan, Wei Ding, Jie Li, Kaifeng Gan, Yandong Shen, Junxiang Xu, Minzhe Zheng

**Affiliations:** Department of Orthopedics, Ningbo Medical Center Lihuili Hospital, No. 1111 Jiangnan Road, Ningbo, 315400 Zhejiang China

**Keywords:** Knee osteoarthritis, ALDH, Oxidative stress, Inflammation, Apoptosis, AQP4

## Abstract

**Background:**

Knee osteoarthritis (KOA) is a common cause of disability among the elderly. We aimed to explore the effects of aldehyde dehydrogenase (ALDH) 2 on the progression of KOA and identifying the potential mechanisms.

**Methods:**

First, ALDH2 expression in knee joint effusion of patients with KOA and the levels of oxidative stress-related markers were determined. After ALDH2 overexpression in monosodium iodoacetate (MIA)-treated SW1353 cells, cell viability was tested with CCK-8 assay. Subsequently, oxidative stress and inflammation-associated factors were measured. Meanwhile, cell apoptosis was assessed with TUNEL staining and expression of apoptosis-related proteins was detected by western blotting. To analyze the mechanism of ALDH2 in KOA, aquaporin 4 (AQP4) expression was determined using western blotting following ALDH2-upregulation. Subsequently, AQP4 was overexpressed to evaluate the changing of oxidative stress, inflammation and apoptosis in SW1353 cells exposed to MIA with ALDH2 overexpression.

**Results:**

Results indicated that knee joint effusion with higher ALDH2 expression displayed lower oxidative stress. In addition, significantly upregulated ALDH2 expression was observed in MIA-treated SW1353 cells. ALDH2 overexpression oxidative stress, inflammation and apoptosis in SW1353 cells exposed to MIA. Moreover, MIA-triggered elevated expression of AQP4, which was reduced by ALDH2 overexpression. By contrast, AQP4-upregulation abrogated the inhibitory effects of ALDH2 on oxidative stress, inflammation and apoptosis in MIA-induced SW1353 cells.

**Conclusions:**

ALDH2 inactivates the expression of AQP4, by which mechanism the MIA-induced oxidative stress, inflammation and apoptosis injuries were alleviated, which provides a novel insight for understanding the mechanism of KOA and a promising target for the treatment of this disease.

## Background

Knee osteoarthritis (KOA) is a common degenerative disease of the musculoskeletal system among the elderly, whose incidence is growing at an alarming rate in recent years with increased population aging [1, 2]. It has been reported that about 14 million Americans are subjected to osteoarthritis of knee [3]. OA represents a major cause of disability and huge social burdens and expenditure. At present, various treatments have been used to treat KOA depending on the severity of this disease [4]. However, no single treatment has proven complete effectiveness. Therefore, it is of great significance to seek for efficient targets for the treatment of KOA.

KOA has been widely accepted as an immune-related disease. Oxidative stress and chronic inflammation are two major driver of the ongoing joint degeneration, which are frequently discussed in the onset and progression of this disease [5]. Aldehyde dehydrogenase 2 (ALDH2), a member of the aldehyde dehydrogenase (ALDH) superfamily that has 19 ALDH subtypes, regulates aldehyde metabolism by eliminating cytotoxic aldehydes, thereby reducing oxidative stress and inhibiting the production of reactive oxygen species (ROS)-related toxic products [6]. Compelling evidence indicate that ALDH2 gene alleviates ketamine-induced cystitis in mouse model through inhibiting oxidative stress [7]. ALDH2 overexpression contributes to the reduction of ROS production and inhibit inflammation in high glucose-induced H9C2 cardiac cell injury [8]. Ausra et al. demonstrated that human articular chondrocytes with strongly expressed ALDH2 have higher expression of COL2A1 and SOX9, which are crucial genes to improve KOA [9]. Therefore, the effects of ALDH2 on KOA has got our research interest. Emerging evidence supports the notion that increasing ALDH2 activity can improve ischemic stroke in rats by downregulating aquaporin 4 (AQP4) expression [10]. It is worthy of note that overexpression of AQP4 in articular chondrocytes exacerbates the severity of adjuvant-induced arthritis in rats [11]. Thus, whether ALDH2 plays a protective role in KOA by regulating AQP4 is a focus of this study.

In the present study, the expression of ALDH2 was determined in knee joint effusion of patients with KOA and monosodium iodoacetate (MIA)-treated chondrocyte. The effects of ALDH2 on oxidative stress, inflammation and apoptosis as well as its regulation on AQP4 were investigated. Collectively, our findings may provide a fresh perspective on therapeutic strategies for KOA.

## Results

### High expression of ALDH2 presents lower oxidative stress in knee joint effusion of patients with KOA

**Fig. 1:**
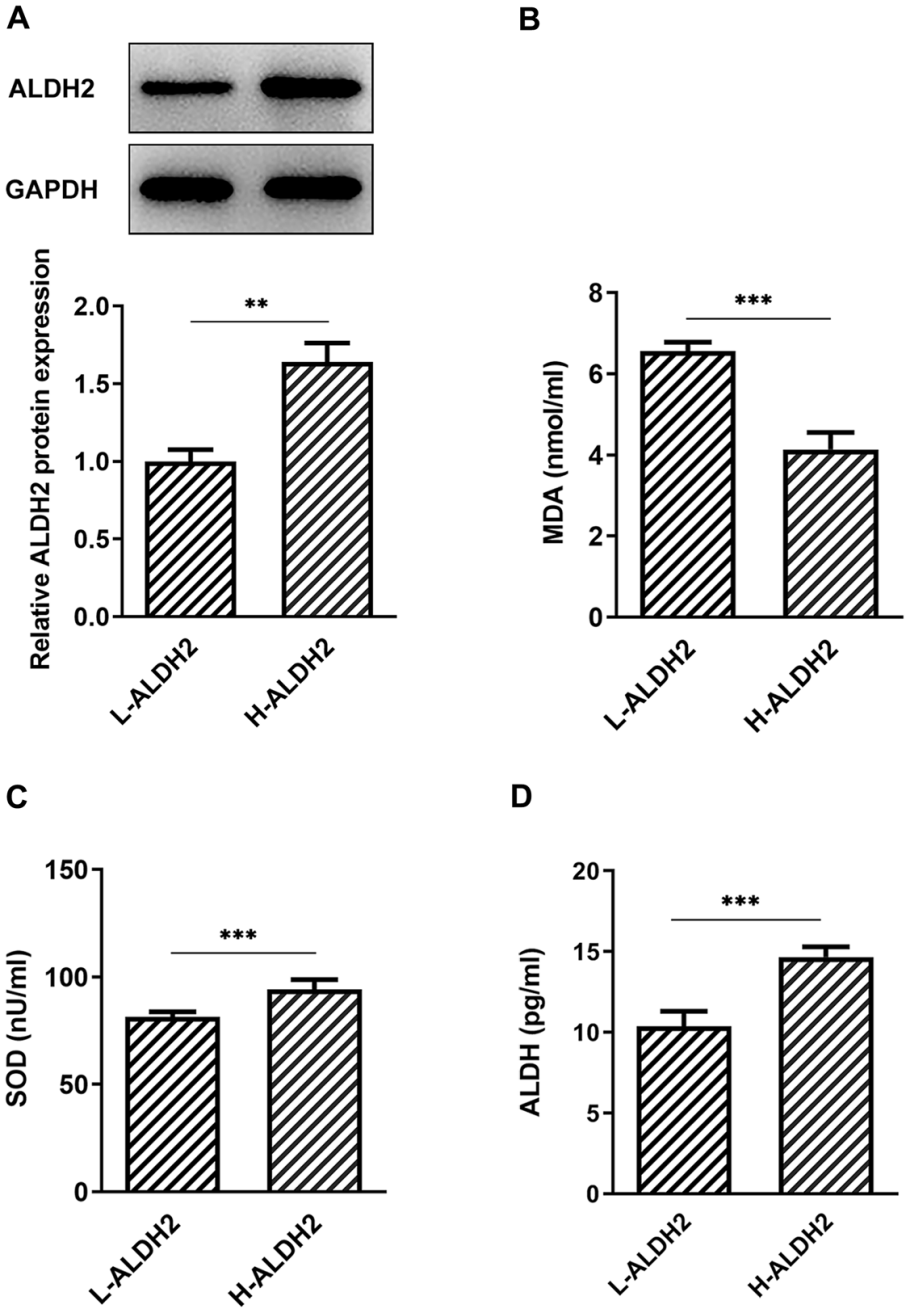
High expression of ALDH2 showed lower oxidative stress in knee joint effusion of patients with KOA. **A** ALDH2 expression was examined using western blot analysis. **B**–**D** Levels of MDA, SOD and ALDH were evaluated with commercial kits. **P < 0.01, ***P < 0.001

First, the knee joint effusion samples of patients with KOA were collected to detect the expression of ALDH2. As displayed in Fig. 1A, ALDH2 expression exhibited different level in different samples. It was found that knee joint effusion with high expression of ALDH2 (H-ALDH2) showed lower level of MDA and higher activities of SOD and ALDH as comparison to that of in the low expression of ALDH2 (L-ALDH2) group (Fig. 1B–D). These data suggest that high expression of ALDH2 presents lower oxidative stress in knee joint effusion of patients with KOA.

### ALDH2 overexpression promotes the proliferation of SW1353 cells exposed to MIA

**Fig. 2:**
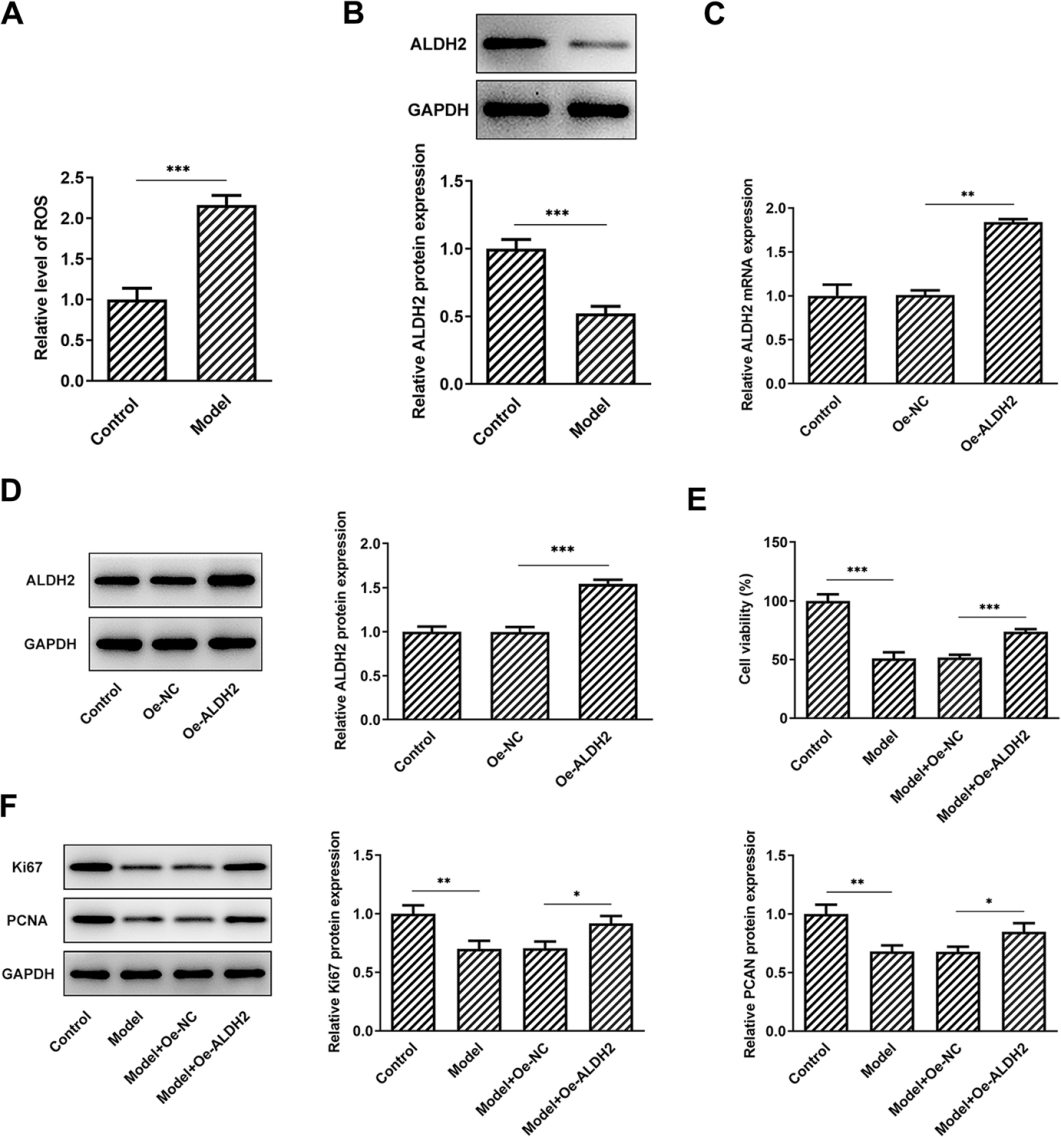
ALDH2 overexpression enhanced the proliferation of SW1353 cells induced by MIA. **A** Level of ROS in MIA-stimulated SW1353 cells was assessed with a commercial kit. **B** Expression of ALDH2 in SW1353 cells exposed to MIA was determined by means of western blot assay. **C**, **D** ALDH2 expression was tested with RT-qPCR and western blotting after transfection. **E** Cell viability was detected by a CCK-8 assay in MIA challenged SW1353 cells with ALDH2 overexpression. **F** Western blot analysis was adopted for the measurement of Ki67 and PCNA expression. *P < 0.05, **P < 0.01, ***P < 0.001

Then, SW1353 cells were induced with MIA to stimulate the KOA model in vitro. A significantly increased ROS content was observed after MIA exposure compared with the untreated group (Fig. 2A). Results of western blot analysis showed that ALDH2 was notably downregulated in the MIA-stimulated SW1353 cells (Fig. 2B). To investigate the role of ALDH2 in the progression of KOA, ALDH2 was overexpressed by transfection with pcDNA 3.1 plasmid, and markedly elevated ALDH2 mRNA and protein expression was found in the Oe-ALDH2 group when compared to the empty vector group (Fig. 2C, D). As what is observable from Fig. 2E, cell viability was conspicuously decreased in the MIA-induced model group, whereas ALDH2-upregulation partially enhanced cell viability as comparison to the model + Oe-NC group. Consistently, MIA exposure led to remarkably reduced expression levels of Ki67 and PCNA compared with the control group (Fig. 2F). By contrast, ALDH2 overexpression relieved the downregulation of Ki67 and PCNA expression induced by MIA. These results provide evidence that ALDH2 is downregulated in MIA-induced SW1353 cells, and gain-function of ALDH2 elevates cell proliferation under this stimulation condition.

### ALDH2 overexpression alleviates MIA-induced oxidative stress and inflammation in SW1353 cells

**Fig. 3:**
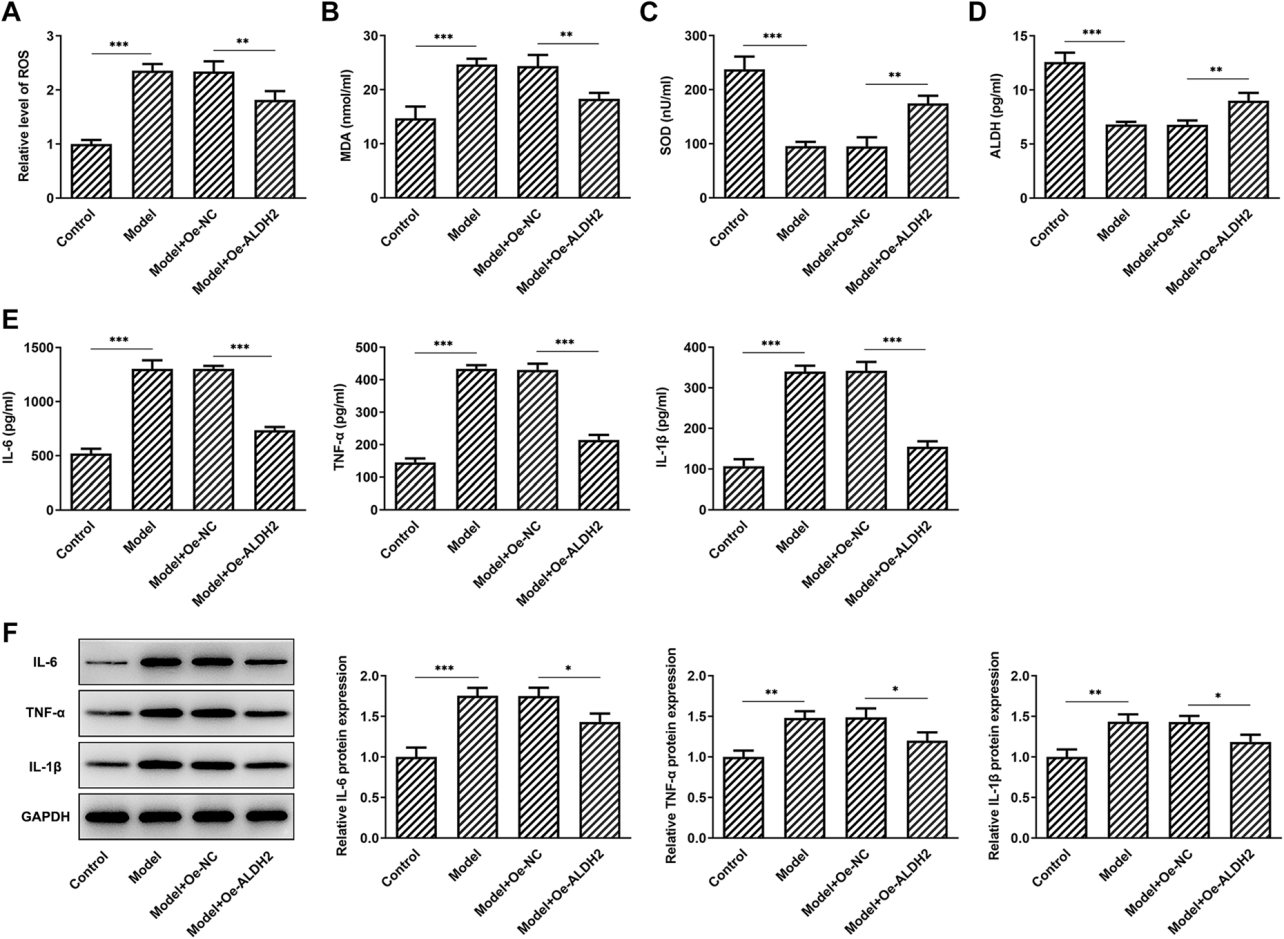
Overexpression of ALDH2 alleviated MIA-induced oxidative stress and inflammation in SW1353 cells. **A**–**D** Levels of ROS, MDA, SOD and ALDH were tested with commercial kits. **E** Contents of IL-6, TNF-α and IL-1β were assessed using ELISA kits. **F** RT-qPCR assay was used to determine the expression of IL-6, TNF-α and IL-1β. *P < 0.05, **P < 0.01, ***P < 0.001

Subsequently, the levels of oxidative stress-related markers were determined using commercial kits. Results presented in Fig. 3A–D indicated that MIA treatment resulted in significant increase in ROS and MDA contents as well as decrease in SOD and ALDH activities compared with the control group. However, ALDH2 overexpression partially attenuated the impact of MIA stimulation on the levels of above-mentioned oxidative stress-associated markers. In addition, as exhibited in Fig. 3E, F, the concentrations of inflammatory factors IL-6, TNF-α, and IL-1β were enhanced apparently after MIA exposure when compared with the control group. On the contrary, as comparison to the empty vector group, gain-function of ALDH2 notably decreased these inflammatory factors. As expected, the mRNA expression of IL-6, TNF-α and IL-1β exhibited the same trend as their concentrations in MIA-induced SW1353 cells. These results potently indicate that ALDH2-upregulation ameliorates MIA-induced oxidative stress and inflammation in SW1353 cells.

### ALDH2 overexpression ameliorates apoptosis of SW1353 cells treated with MIA

**Fig. 4:**
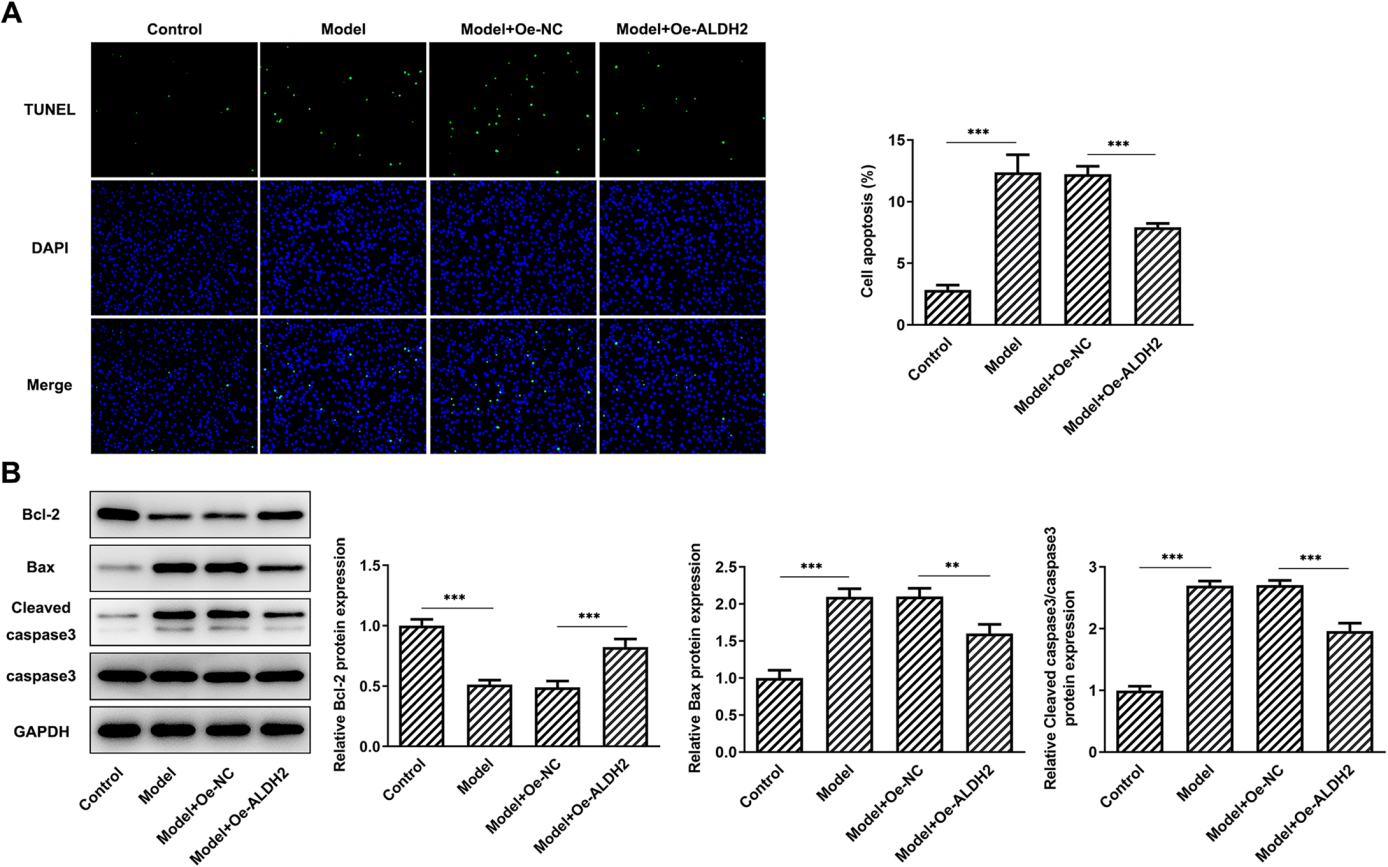
ALDH2 overexpression ameliorated apoptosis of SW1353 cells after MIA stimulation. **A** TUNEL staining was used to determined cell apoptosis. Magnification, × 200. **B** Expression of apoptosis-related proteins was tested using western blot analysis. **P < 0.01, ***P < 0.001

Cell apoptosis was evaluated by means of TUNEL staining. Results in Fig. 4A suggested that the rate of cell apoptosis was significantly enhanced after MIA stimulation when compared to the control group. Concurrently, as comparison to the Model + Oe-NC group, gain-function of ALDH2 notably reduced cell apoptosis. Besides, MIA led to remarkable downregulated Bcl-2 expression and upregulated Bax and cleaved caspase3 compared with the control, which was partially restored by ALDH2 overexpression (Fig. 4B). These findings indicate that overexpression of ALDH2 attenuates apoptosis of SW1353 cells after MIA challenge.

### AQP4 overexpression blocks the inhibitory effects of ALDH2-upregulation on the oxidative stress and inflammation in MIA-induced SW1353 cells

**Fig. 5:**
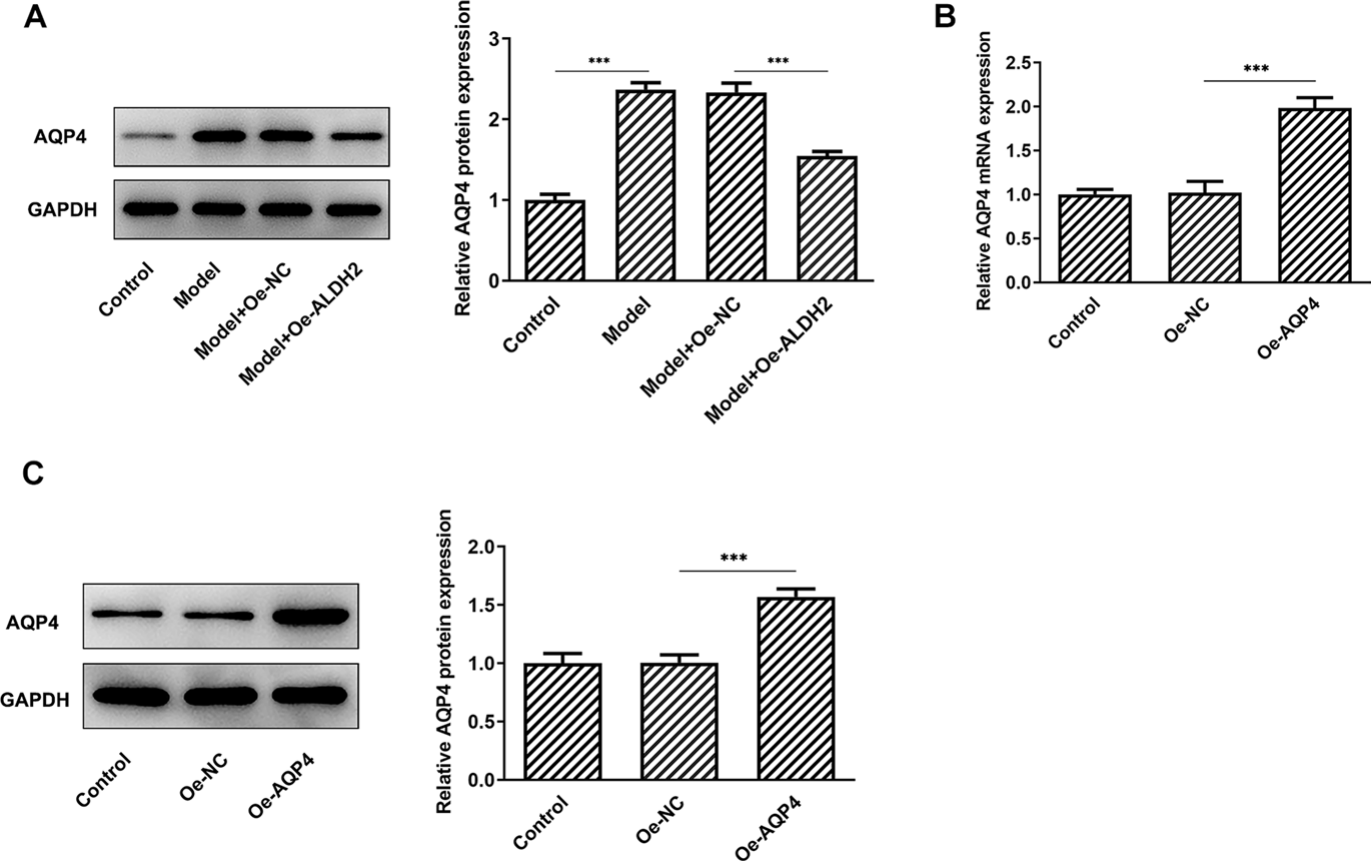
ALDH2 inhibited AQP4 expression in MIA-induced SW1353 cells. **A** Expression of AQP4 was determined with western blot analysis after ALDH2 overexpression in MIA-induced SW1353 cells. **B**, **C** RT-qPCR and western blot assay were used to assess AQP4 expression after transfection with AQP4 plasmid in SW1353 cells. *P < 0.05, ***P < 0.001

**Fig. 6:**
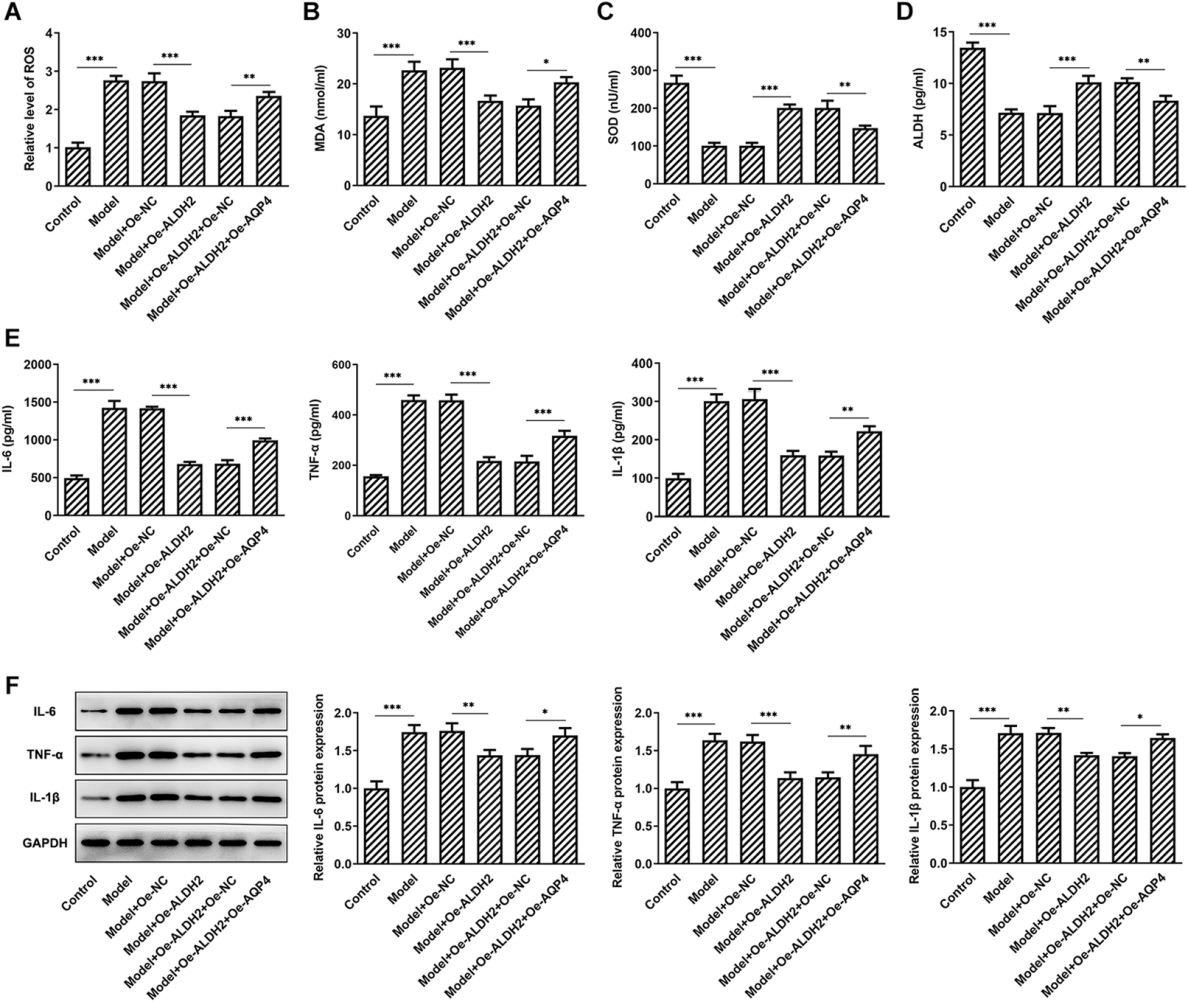
AQP4 overexpression attenuated the inhibitory effects of ALDH2 overexpression on the oxidative stress and inflammation in MIA-induced SW1353 cells. **A**–**D** Levels of ROS, MDA, SOD and ALDH were tested with commercial kit. **E**–**F** Levels of IL-6, TNF-α and IL-1β were, respectively, evaluated by means of ELISA kits and western blot analysis. *P < 0.05, **P < 0.01, ***P < 0.001

AQP4 expression was tested by western blot analysis. As displayed in Fig. 5A, MIA stimulation conspicuously elevated AQP4 expression when compared to the control group. However, a significant decrease in AQP4 expression was observed after ALDH2 overexpression. Afterwards, AQP4 was overexpressed by transfection with AQP4 plasmid and markedly enhanced AQP4 mRNA and protein levels were found compared with the empty control group in Fig. 5B, C. As what is observable from Fig. 6A–D, AQP4-upregulation increased the levels of ROS and MDA, coupled with decreased activities of SOD and ALDH when compared to the SW1353 cells transfection with ALDH2 and empty plasmids under MIA treatment condition. In addition, the contents and protein expression of IL-6, TNF-α and IL-1β were partially intensified after AQP4 overexpression in MIA-induced SW1353 cells with ALDH2-upregulation (Fig. 6E, F). These observations reveal that ALDH2 inhibits oxidative stress and inflammation in MIA challenged SW1353 cells via inhibiting AQP4 expression.

### AQP4 overexpression reduced the impact of ALDH2-upregulation on the apoptosis of SW1353 cells exposed to MIA

**Fig. 7:**
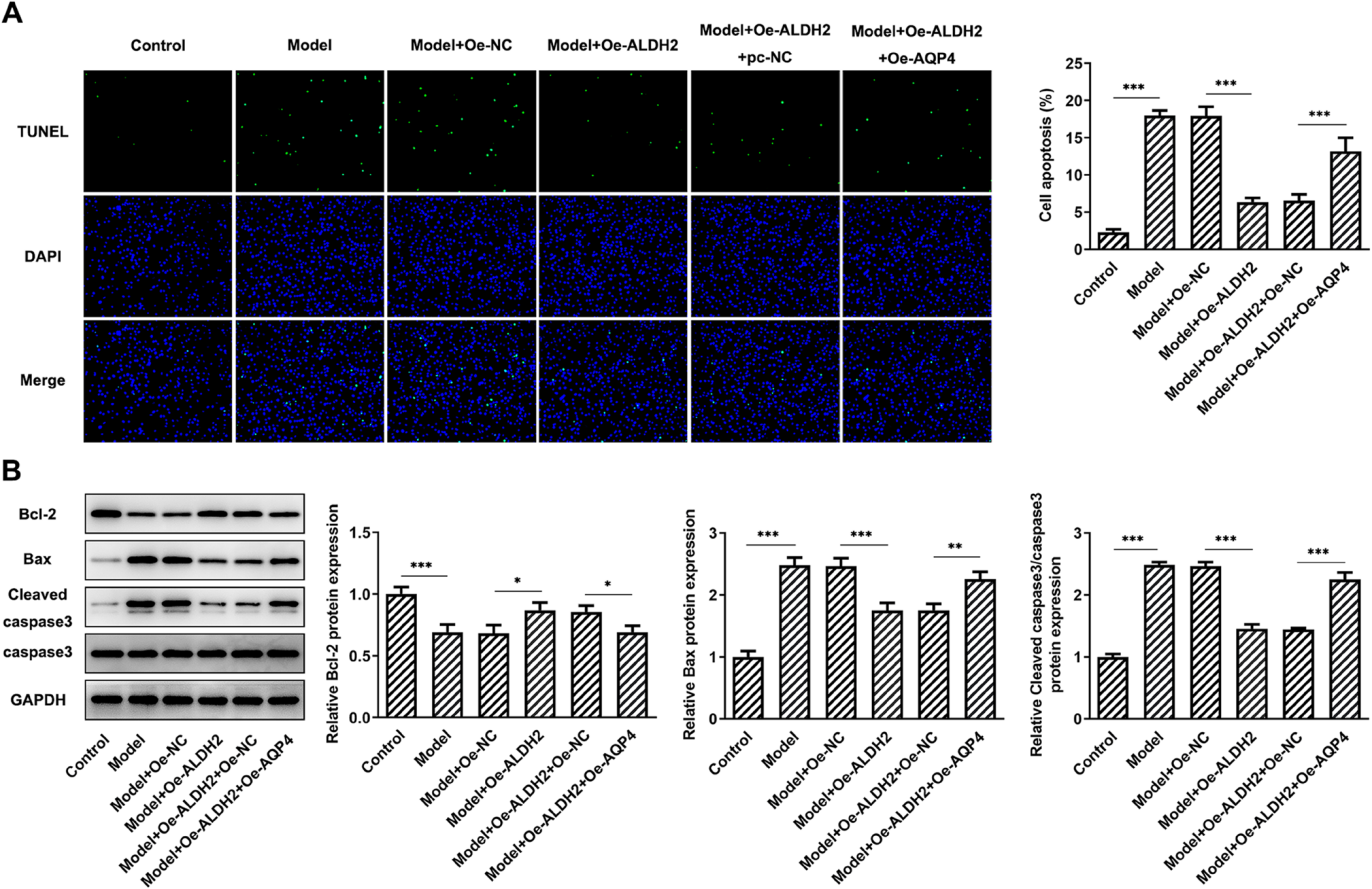
AQP4 overexpression reduced the impact of ALDH2-upregulation on the apoptosis of SW1353 cells exposed to MIA. **A** TUNEL staining was employed to evaluate cell apoptosis. Magnification, × 200. **B** Expression of apoptosis-related proteins was detected using western blot analysis. *P < 0.05, **P < 0.01, ***P < 0.001

According to the result of TUNEL assay (Fig. 7A), increased number of apoptotic SW1353 cells was found in the AQP4 overexpressed group as comparison to the Model + Oe-ALDH2 + Oe-NC group. Furthermore, gain-function of AQP4 resulted in reduced Bcl-2 expression accompanied by elevated Bax and cleaved caspase3 expression when compared to the MIA-induced SW1353 cells with ALDH2-upregulation (Fig. 7B). Through the above findings we proved that ALDH2 alleviates MIA-induced apoptosis in SW1353 cells via inhibiting AQP4 expression.

## Discussion

KOA is a degenerative disease characterized by progressive loss of articular cartilage in knee joints, potentially resulting in disability in the aging population [12]. The human chondrosarcoma cell line (SW1353) has been widely used for the investigation of KOA [13]. In cultures, chondrocyte treated with MIA, an inhibitor of glyceraldehyde-3-phosphate dehydrogenase that can cause chondrocyte death, is the generally accepted cell model to stimulate the progression of KOA in humans [14–16]. In this study, we demonstrated that highly expression of ALDH2 in human KOA knee joint effusion is closely related to the decreased oxidative stress. ALDH2-upregulation alleviates the oxidative stress, inflammation and apoptosis in MIA-induced SW1353 cells. Mechanically, the effects of ALDH2 on the progression of KOA might mediated by inhibiting AQP4 expression.

A considerable body of evidence indicates that multiple factors including bone loss and subchondral densification contribute to the pathophysiological process of KOA. Among these factors, chronic inflammation and excessive oxidative stress dysregulates genetic and epigenetic pathways to inhibit chondrocytic activity and cartilage homeostasis in the KOA microenvironment [17]. The involvement of oxidative stress into both KOA pathogenesis and the effects of therapeutic agents applied in KOA cases has already been confirmed [18–20]. During oxidative stress, high ROS levels trigger lipid peroxidation and the formation of an end-product of oxidation MDA, which influences the activities of antioxidant enzymes, such as SOD and ALDH [21, 22]. Increasing evidence suggests that pro-inflammatory cytokines, such as IL-6, TNF-α and IL-1β, release from the inflammatory synovial tissue and synovial fluid to inhibit chondrocyte proliferation, which induces the apoptosis of chondrocyte and ultimately results in articular cartilage degeneration [23, 24]. A clinical trial performed in patients of KOA demonstrated that curcuma longa extract administration brings significant clinical improvement the attenuation of oxidative stress and inflammation [25]. ALDH2, a mitochondria enzyme, can not only detoxify acetaldehyde derived from ethanol metabolism in the liver but also responsible for detoxification of other reactive aldehydes derived from endogenous metabolism in other tissues [26]. Clinically, abnormal ALDH2 expression contributes to a large amount of human diseases [27, 28]. It has been reported that ALDH2 knock-out mice exhibits significantly enhanced oxidative stress and inflammation in ketamine-induced cystitis [7]. Activation of ALDH2 protects against fibrosis, apoptosis and necroptosis in primary cardiomyocytes induced by high glucose via repression of oxidative stress and inflammation [29]. Importantly, ALDH2 mutation promotes skeletal muscle atrophy in mice by accumulation of oxidative stress [30]. Aldehyde-stress resulting from ALDH2 mutation accelerates osteoporosis due to impaired osteoblastogenesis [31]. It is worthy of note that human articular chondrocytes with strongly expressed ALDH2 have higher expression of COL2A1 and SOX9, which are crucial genes to improve KOA [9]. In the present study, high expression of ALDH2 was related to lower oxidative stress in knee joint effusion of patients with KOA, and ALDH2-konckdown promoted proliferation as well as inhibited oxidative stress, inflammation and apoptosis in MIA-induced SW1353 cells.

Research has proposed that increasing ALDH2 activity can improve ischemic stroke in rats via inactivation of AQP4 expression [10]. AQP4 is a member of the water channel family, whose downregulation plays a neuroprotective role in middle cerebral artery occlusion rats by repressing oxidative stress and inflammatory response [32]. The excess production of ROS can promote the expression of AQP4, and inhibition of AQP4 reduces lung inflammation in irradiation-induced mice [33, 34]. Emerging evidence supports the notion that AQP4 overexpression in articular chondrocytes exacerbates the severity of adjuvant-induced arthritis in rats [11]. Extrapolating from the above findings, we presumed the protective effects of ALDH2 in KOA in vitro model is mediated by inhibiting AQP4 expression. In addition, the present study is the first to explore the regulatory effects of ALDH2 and AQP4 on the progression of KOA. We found that gain-function of ALDH2 downregulated AQP4 expression, which was in according with the previous study [10]. Furthermore, AQP4 overexpression reversed the attenuation of oxidative stress, inflammation and apoptosis triggered by ALDH2-upregulation in SW1353 cells exposed to MIA.

## Conclusions

Taken together, findings in this study is the first to demonstrate the protective effects of ALDH2 on the progression of KOA in a MIA-treated chondrocyte model. ALDH2 alleviates MIA-induced oxidative stress, inflammation and apoptosis in SW1353 by inactivating AQP4 expression. Data in this study reveal the roles of ALDH2 and AQP4 in KOA, which provides novel insights into the mechanism of KOA physiology and new strategies for developing therapeutic interventions. However, the conclusion of this study only came from in vitro cell model, lacking the validation of in vivo animal models, which is a limitation of this study. Our future research will aim to make the conclusion of this study stronger using animal model and uncover the underlying mechanisms.

## Materials and methods

### Sample collection

The human KOA knee joint effusion samples were collected from patients (*n* = 16; ten women and six men, aged 42–74 years) undergoing knee replacement surgery or arthroscopy at Ningbo Medical Center Lihuili Hospital (Ningbo, China). None of the patients had received intra-articular steroid injections within 3 months prior to surgery. This study was approved by the ethics committee of Ningbo Medical Center Lihuili Hospital (Ningbo, China). Written informed consent was obtained from each patient.

### Cell culture

The human chondrosarcoma cell line (SW1353) was provided by the (ATCC^®^ HTB-94™) were purchased from the American Type Culture Collection (Rockville, MD, USA). The cells were grown in Dulbecco’s modified Eagle’s medium (DMEM; Gibco, Grand Island, USA) containing 10% fetal bovine serum (FBS; HyClone, Auckland, NZ, USA). The incubator was set as 5% CO_2_ humidified atmosphere at 37 °C. Attached cells were treated with 5 μM MIA (Sigma-Aldrich, St Louis, MO, USA) for 24 h to simulate the KOA model in vitro according to a previous study [14].

### Cell transfection

SW1353 cells were seeded at 2 × 10^5^ cells/well into 6-well plates and cultured at 37 °C until they reached 80% confluence. pcDNA 3.1 containing ALDH2 (Oe-ALDH2) or AQP4 (Oe-AQP4), and the corresponding empty vectors (Oe-NC) were synthesized by Shanghai GenePharma Co., Ltd. Cells were transfected with the respective plasmids using Lipofectamine^®^ 2000 reagent (Invitrogen; Thermo Fisher Scientific, Inc.), according to the manufacturer’s instructions. After 48 h transfection, cells were collected and the transfection efficiency was assessed via reverse transcription-quantitative (RT-q) PCR and western blot analyses.

### Cell viability assay

Cell viability was evaluated by means of a Cell Counting Kit-8 (CCK-8; Shanghai YiSheng Biotechnology Co., Ltd., Shanghai, China). The transfected SW1353 cells were seeded into 96-well plates (3000 cells/well) and incubated for 48 h. Then, 10 μL CCK-8 working solution was added to each well and incubated for another 4 h at 37 °C. The optical density (OD) was evaluated at the 450 nm wavelength using a microplate reader (Bio-Rad Laboratories, Inc.).

### Measurement of oxidative stress

The knee joint effusion were centrifuged and the supernatant was obtained to evaluate the contents of malondialdehyde (MDA) and superoxide dismutase (SOD). In addition, after removal of cells and debris via centrifugation, the supernatant was then collected. The production of ROS and MDA as well as the activities of SOD and ALDH were monitored with the corresponding kits according to the manufacturers’ instructions. Above-mentioned kit were all purchased from Nanjing Jiancheng Bioengineering Institute (Nanjing, China). Subsequently, the absorbance was measured using a microplate reader (Bio-Rad Laboratories, Inc.).

### Determination of the levels of inflammatory factors

Enzyme-linked immunosorbent assay (ELISA) was used to examine the levels of inflammatory factors including interleukin-6 (IL-6), interleukin-1β (IL-1β) and tumor necrosis factor α (TNF-α) in the cell culture medium. The levels of the inflammatory factors were measured according to the manufacturer's instructions (Shanghai Xitang Biotechnology Co., Ltd.). The optical density values at 450 nm were read on a plate reader (BioTek Instruments, Winooski, VT, USA).

### TUNEL staining

For the evaluation of apoptosis, a TUNEL Apoptosis Detection kit (Invitrogen, Carlsbad, CA, USA) was employed in this study following manufacturer's recommendations. SW1353 cells were fixed with 4% paraformaldehyde and then incubated with 0.1% Triton X-100 to permeabilize the cell membrane. Cells were then incubated with 50 μL TUNEL reaction buffer for 1 h at 37 °C. The nuclei were counterstained with DAPI in the dark and the slides were then mounted with anti-fade mounting medium. Images were visualized and captured using a fluorescence microscope (Olympus Corporation).

### RT-qPCR

Total RNA was extracted from cartilage tissues and chondrocytes cells utilizing TRIzol^®^ reagent (Invitrogen; Thermo Fisher Scientific, Inc.). Complementary DNA was then synthesized by means of the Prime Script™ RT Master Mix (TaKaRa Bio) in accordance with the manufacturer’s protocol. Subsequently, using cDNA as the template, the gene expression levels were analyzed via qPCR, which was conducted using iTaq™ Universal One-Step iTaq™ Universal SYBR^®^ Green Supermix (Bio-Rad Laboratories, Inc.) on an ABI 7500 instrument (Applied Biosystems; Thermo Fisher Scientific, Inc.). Primers used in this study were designed and synthesized by Shangon Company (Shanghai, China). GAPDH were used as internal controls for normalization. Gene expression levels were quantified according to the 2^−ΔΔCt^ method [35].

### Western blot analysis

Total proteins were extracted from cells using RIPA lysis buffer (Beyotime Institute of Biotechnology). A bicinchoninic acid protein assay kit (Beyotime Institute of Biotechnology) was employed to test the protein concentration. Afterwards, normalized volumes of samples (40 µg protein per lane) was isolated on SDS-PAGE on a 10% gel and transferred onto PVDF membranes. Subsequently, membranes were blocked with 5% non-fat milk, prior to incubation with primary antibodies for the target proteins. Following incubation with the goat anti-rabbit horseradish peroxidase-conjugated secondary antibodies, the bands were visualized using an Odyssey Infrared Imaging Scanner (LI-COR Biosciences). The intensities of protein bands were quantified by Image J software and the relative protein level was normalized to GAPDH.

### Statistical analysis

Data were obtained from three independent experiments and are presented as the mean ± standard deviation. Statistical analysis was illustrated with GraphPad Prism (version 8.0; GraphPad Software, Inc.). Comparisons between two groups were evaluated using Student’s *t* test. Comparisons involving multiple samples were analyzed by one-way analysis of variance (ANOVA) followed by Tukey’s post hoc test. *P* < 0.05 was accepted as statistically significant.

## Data Availability

All data generated or analyzed during this study are included in this published article.
